# Not all LGE is the same. Scar contrast volume of distribution is lower in HCM than in infarction

**DOI:** 10.1186/1532-429X-14-S1-O95

**Published:** 2012-02-01

**Authors:** Thomas A Treibel, Steven K White, Daniel Sado, Jonathan Hasleton, Andrew Flett, Anna S Herrey, Derek J Hausenloy, James Moon

**Affiliations:** 1 Department of Cardiology, The Heart Hospital, University College London Hospitals NHS Trust, London, UK; 2 The Hatter Cardiovascular Institute, University College London Hospitals NHS Trust, London, UK

## Background

Focal fibrosis is visible as late gadolinium enhancement when using inversion recovery (IR) imaging late after a contrast bolus and carries major clinical import. The IR technique results in high sensitivity to regional T1 variation but sacrifices absolute quantification meaning that differing degrees of focal interstitial expansion will look the same (bright).

We hypothesized that LGE in different disease processes would have different degrees of interstitial expansion. We used equilibrium contrast CMR (EQ-CMR)^1^, to measure the degree of interstitial expansion by measuring the volume of distribution of contrast, Vd(m).

## Methods

**Figure 1:**
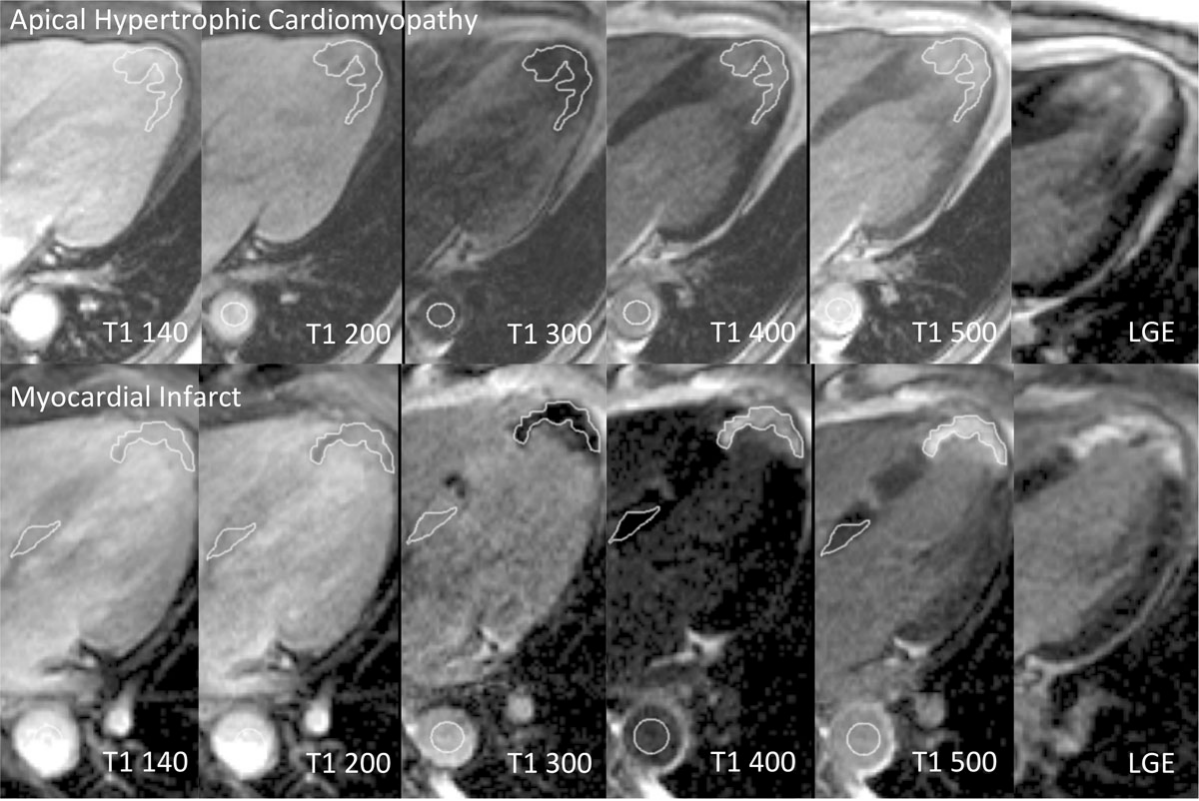
FLASH T1 mapping in apical HCM and MI with regions of interest drawn in the blood (aorta), areas of late gadolinium enhancement (LGE) and remote myocardium in MI.

A prospective study of patients with HCM (n=58, n=16 with LGE in the interrogated slice) and acute myocardial infarction (n=19), who had LGE and EQ-CMR within the region of LGE. T1 of blood and myocardium were measured using multi flip angle T1 FLASH IR maps at increasing inversion time before and after reaching contrast equilibrium [1]. Regions of interest were drawn in the aorta, remote myocardium and myocardium with LGE (see Figure 1).

**Figure 2:**
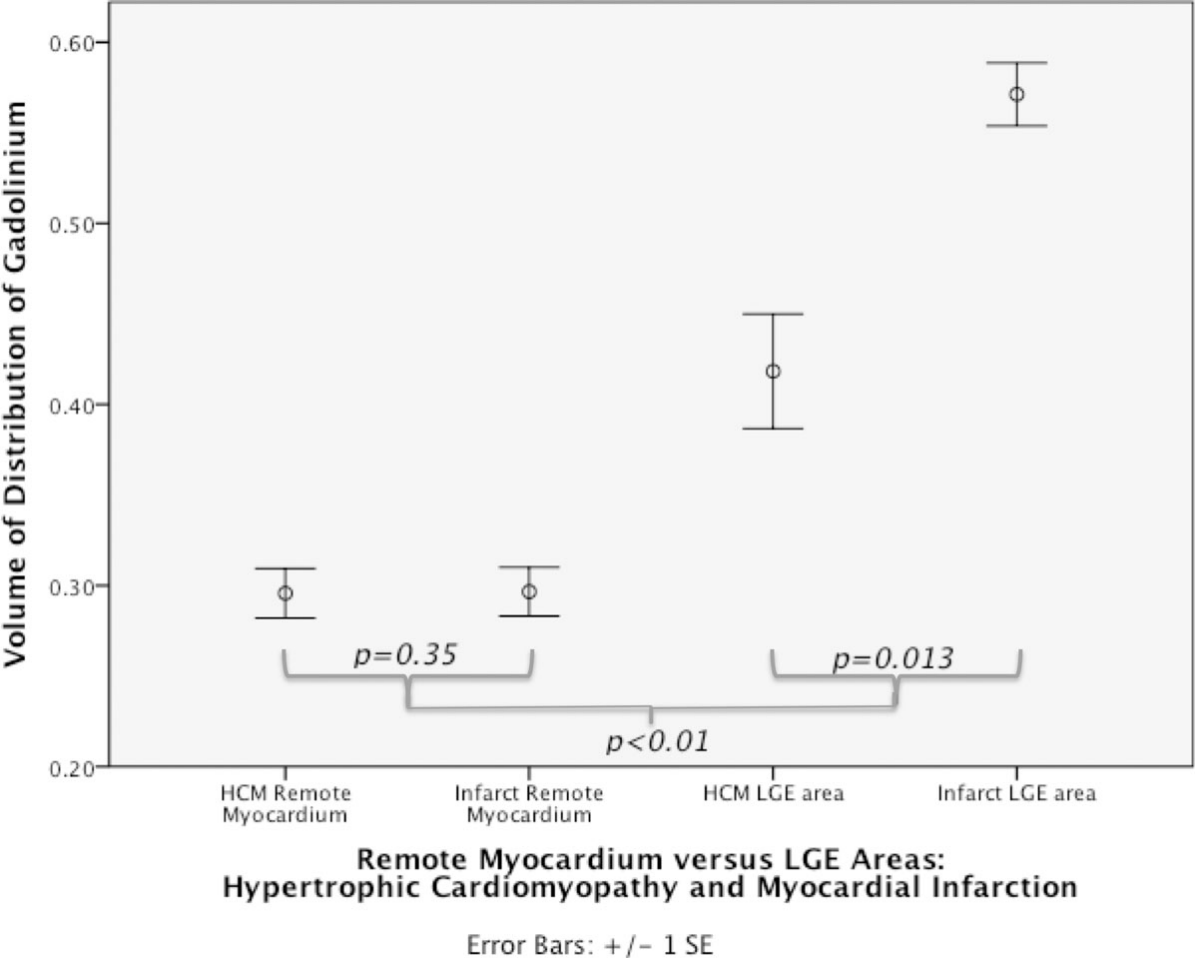


## Results

HCM LGE had a lower Vd(m) than infarct LGE (0.43 vs 0.56; *p*=*0.013*). There was no difference between remote myocardial Vd(m) between the diseases (0.30 vs 0.33; *p*=*0.35*).

## Conclusions

LGE in HCM has less interstitial expansion than LGE in infarction. EQ-CMR allows differentiation of LGE in degrees of interstitial expansion and, by inference, degrees of myocyte loss. The presence of residual myocytes within HCM LGE may be of biological interest, potentially representing, for example, an arrhythmic substrate.

## Funding

British Heart Foundation; National Institute for Health Research.

## References

[B1] Flett AS Circulation 2010 122 138 44 10.1161/CIRCULATIONAHA.109.930636 20585010

